# Intense interval training in healthy older adults increases skeletal muscle [^3^H]ouabain‐binding site content and elevates Na^+^,K^+^‐ATPase α_2_ isoform abundance in Type II fibers

**DOI:** 10.14814/phy2.13219

**Published:** 2017-04-03

**Authors:** Victoria L. Wyckelsma, Itamar Levinger, Robyn M. Murphy, Aaron C. Petersen, Ben D. Perry, Christopher P. Hedges, Mitchell J. Anderson, Michael J. McKenna

**Affiliations:** ^1^ Clinical Exercise Science Research ProgramInstitute of Sport, Exercise and Active Living (ISEAL)VictoriaAustralia; ^2^ Department of Biochemistry and GeneticsLa Trobe Institute for Molecular ScienceLa Trobe UniversityVictoriaAustralia; ^3^ Renal DivisionDepartment of MedicineEmory UniversityAtlantaGeorgia; ^4^ Baker IDI Heart and Diabetes InstituteMelbourneAustralia

**Keywords:** Aging, exercise, Na^+^,K^+^‐pump, potassium, single fiber

## Abstract

Young adults typically adapt to intense exercise training with an increased skeletal muscle Na^+^,K^+^‐ATPase (NKA) content, concomitant with reduced extracellular potassium concentration [K^+^] during exercise and enhanced exercise performance. Whether these changes with longitudinal training occur in older adults is unknown and was investigated here. Fifteen older adults (69.4 ± 3.5 years, mean ± SD) were randomized to either 12 weeks of intense interval training (4 × 4 min at 90–95% peak heart rate), 3 days/week (IIT, *n* = 8); or no exercise controls (*n* = 7). Before and after training, participants completed an incremental cycle ergometer exercise test until a rating of perceived exertion of 17 (very hard) on a 20‐point scale was attained, with measures of antecubital venous [K^+^]_v_. Participants underwent a resting muscle biopsy prior to and at 48–72 h following the final training session. After IIT, the peak exercise work rate (25%), oxygen uptake (16%) and heart rate (6%) were increased (*P* < 0.05). After IIT, the peak exercise plasma [K^+^]_v_ tended to rise (*P* = 0.07), while the rise in plasma [K^+^]_v_ relative to work performed (nmol.L^−1^.J^−1^) was unchanged. Muscle NKA content increased by 11% after IIT (*P* < 0.05). Single fiber measurements, increased in NKA α_2_ isoform in Type II fibers after IIT (30%, *P* < 0.05), with no changes to the other isoforms in single fibers or homogenate. Thus, intense exercise training in older adults induced an upregulation of muscle NKA, with a fiber‐specific increase in NKA α_2_ abundance in Type II fibers, coincident with increased muscle NKA content and enhanced exercise performance.

## Introduction

Sustained skeletal muscle contractions require repeated action potential (AP) propagation along sarcolemmal and t‐tubular membranes. During exercise, the cellular Na^+^ influx and K^+^ efflux can be large and lead to muscle membrane depolarization, inexcitability and fatigue, and while well established in in vitro models (Sejersted and Sjøgaard 2000; Dutka and Lamb 2007; McKenna et al. 2008), this has been difficult to establish directly in contracting human skeletal muscle (McKenna et al. 2008).

The Na^+^,K^+^‐ATPase (NKA) protein is critical in regulating these Na^+^/K^+^ fluxes in skeletal muscle and thus acts to preserve muscle membrane excitability and contractile function (McKenna et al. 2008; Clausen 2013). In human skeletal muscle, three NKA isoforms are expressed for each of the catalytic α (α_1‐3_) and the regulatory β (β_1‐3_) subunits (Murphy et al. 2004), with a *γ* subunit known as FXYD1 is an important regulator of NKA activity (Bibert et al. 2008). In rodent skeletal muscle, the α_1_ isoform is important for basal Na^+^/K^+^ regulation, whereas the α_2_ isoform is primarily responsible for regulating the large Na^+^/K^+^ fluxes during muscle contraction (Radzyukevich et al. 2013; Manoharan et al. 2015) and is the most abundant α isoform (Hansen 2001; He et al. 2001); the role for the α_3_ isoform in skeletal muscle is unclear. The β_1_ isoform is critical in NKA integration into the cell membrane (Geering 2001) and plays a key role in regulating NKA enzymatic activity (Lavoie et al. 1997; Blanco and Mercer 1998).

The NKA content can be measured in human muscle by the [^3^H]ouabain‐binding site content, since ouabain binds with high affinity to the α isoforms in human cells (Nørgaard et al. 1984; Wang et al. 2001). In young adults, the muscle NKA content, α and β isoforms are highly adaptable to high‐intensity physical exercise training (Mohr et al. 2007; Iaia et al. 2008; Bangsbo et al. 2009). Typically, in young adults, the NKA content is increased by around 15% with different training types (McKenna et al. 1996). Older adults who had trained for 12–17 years had a 30–40% higher muscle NKA content than untrained older adults (Klitgaard and Clausen 1989); but as this was a cross‐sectional study, it remains unclear whether this substantial difference was due to training per se. In aged rats, fiber‐type specific increases were found after endurance training for the NKA α_1_, α_2_ and β_1_ isoforms in red and white muscles (Ng et al. 2003). The functional implications of upregulated muscle NKA content include an enhanced capacity to regulate Na^+^/K^+^ fluxes across muscle membranes during contractions, with improved K^+^ regulation and reduced fatigability (Nielsen et al. 2004; McKenna et al. 2008). Little is known about the training effects on K^+^ regulation during exercise in older adults. A cross‐sectional study reported a greater rate of rise in venous K^+^ concentration [K^+^] during exercise in older than young adults (Ford et al. 1993); this might reflect underlying muscle NKA differences, since the terbutaline‐induced K^+^ uptake into forearm muscle was reduced in the aged (Ford et al. 1995).

Aging is associated with a gradual loss of muscle mass and strength (Deschenes 2004) which may be attributed to alterations of both muscle and motor unit morphology (Hunter et al. 2016). These affect muscle fiber‐type and recruitment of these muscle fibers. To date, there are conflicting results in regard to the effects of aging on NKA in human skeletal muscle. Muscle NKA content did not differ between aged and young adults in three studies (Klitgaard and Clausen 1989; McKenna et al. 2012; Wyckelsma et al. 2016), while another found a lower content in individuals aged 68–81 years compared to 55–68 years (Perry et al. 2013). We recently reported that with aging the muscle NKA α_1_ isoform was upregulated in Type I fibers (Wyckelsma et al. 2016). Skeletal muscle is heterogeneous in nature, in broad terms being comprised of slow‐twitch, oxidative (Type I) and/or fast‐twitch oxidative or glycolytic (Type II) muscle fibers. The proportion of a given fiber‐type is dependent on, among other factors, an individual's age as there is a progressive decline in Type II fibers with age (Evans and Lexell 1995). Any differences in the extent of fiber loss or transition between fiber types may contribute to the conflicting results for NKA content in whole muscle homogenates from aged individuals. The sensitivity of current methods prevents the determination of muscle NKA content in individual fibers, as measured by [^3^H]ouabain‐binding site content. However, the measurement of NKA isoform protein abundances in single muscle fibers is possible (Thomassen et al. 2013; Wyckelsma et al. 2015, 2016). In particular, such measurements provide vital information when conducted alongside whole muscle homogenates, which will contain a mixed fiber population of unknown proportions, in muscle obtained from elderly individuals.

High intensity interval training (HIT) is time‐efficient and easily implemented (Gibala et al. 2012) and induces marked muscle metabolic adaptations in young adults (Gibala et al. 2012; Cochran et al. 2014; Gillen et al. 2014), and middle‐aged individuals (Levinger et al. 2014). However, the effect of intense interval training (IIT) on NKA content and isoforms in older adults is not clear. We therefore tested the hypothesis that muscle NKA content, as well as key NKA isoforms in whole muscle homogenates and in single fibers will increase with intense exercise training in older adults. We also hypothesized that training in older adults will enhance exercise performance associated with a reduction in plasma [K^+^] during exercise. We also explored the suitability of IIT for training apparently healthy older adults.

## Methods

### Participants

Twenty older adults volunteered and gave signed informed consent for the study; 17 passed the initial prescreening assessments and 15 of these participants completed the study. All participants gave written informed consent in accordance with the Declaration of Helsinki. The protocol was approved by the Victoria University Human Research Ethics committee. Potential participants were excluded from the study if they had any of the following conditions: Diabetes (Type I or II), chronic heart disease, severe hypertension (systolic 160–179 mmHg systolic; diastolic 100–109 mmHg), severely overweight/obese (BMI > 30), an uncontrolled metabolic disease (such as uncontrolled diabetes) and/or cardiovascular disease. Participants with any pre‐existing injury impeding their ability to perform exercise were also excluded from the study. Finally, the final exclusion criteria included those who were on medications known to affect the NKA, including salbutamol (5). Both males and females were included as our group has previously published no sex differences in skeletal muscle NKA content in healthy young adults (Murphy et al. 2007).

Physical characteristics of the 15 older adults who completed the study (nine male, six female) who completed the study were; age 69.4 ± 3.5 years, height 170.8 ± 10.4 cm, body mass 75.2 ± 13.0 kg and BMI 21.6 ± 2.6 kg.m^−2^ (mean ± SD); their self‐reported physical activity duration was 8.2 ± 2.6 h per week. The questionnaire asked participants about the amount of exercise performed in the previous 7 days and included all activities ranging from structured exercise through to incidental exercise including household tasks. Each category was graded into a different category of perceived difficulty, which ranged from moderate, to hard and to very hard. The importance of recruiting older adults as participants who maintain physical activity has recently been published (Lazarus and Harridge 2016). Our recruitment of active older adults allowed us to examine the direct effects of IIT in an older population to minimize possible confounding effects of prolonged sedentary behaviors on muscle in addition to adverse effects of aging per se. The study was approved by the Victoria University Human Research Ethics Committee and conforms to the Declaration of Helsinki.

### Experimental overview

Participants were randomized into either intense‐interval training (IIT, *n* = 8, two females, six males) or a no exercise control group (CON, *n* = 7, four females, three males) undertook an initial familiarization session comprising a sign‐ and symptom‐limited incremental exercise test on a cycle ergometer. On a separate day participants underwent a resting muscle biopsy and repeated the incremental exercise test with venous blood sampling to measure plasma [K^+^], and with measurement of the peak exercise heart rate (HR_peak_) to determine target heart rate for training sessions. The muscle biopsy and incremental exercise test with blood sampling were repeated following the IIT or CON period. The post‐training biopsy was taken 48–72 h following the final training session and the exercise test was performed 24–48 h following the biopsy.

### Sign‐ and symptom‐limited incremental exercise test

The protocol for the familiarization and invasive incremental exercise tests were identical except that a shortened test was conducted for the familiarization session. Exercise tests were conducted on an electronically braked cycle ergometer (Excalibur Sport, Lode, The Netherlands) and blood pressure was measured before and during exercise by auscultation using a standard mercury sphygmomanometer. Participants commenced cycling at 20 W at a cadence between 50 and 70 revolutions per minute (rpm), with workrate increased every min by 20 W for males and 10 W for females. To minimize potential adverse risks, each exercise test was sign‐ and symptom‐limited, and was continued until a symptom‐limited endpoint, defined for the familiarization as when participants advised a rating of perceived exertion (RPE) of 13, corresponding to a reading of “somewhat hard”, and for the experimental test as when a RPE of 17 (“very hard”) from a 20‐point scale (Borg 1982) was attained. Participants were asked to indicate their RPE at 45 sec of each minute during exercise; when an RPE of 17 was indicated participants were then asked to complete the final 15 sec of that exercise bout. Before, during and after the experimental test cardiac rhythm and HR were continuously monitored via a 12‐lead electrocardiogram. The highest heart rate achieved during peak exercise (at RPE 17) was defined as the HR_peak_. Blood pressure was taken manually at rest, throughout exercise and for up to 10 min of recovery.

During exercise, participants breathed through a Hans‐Rudolph 3‐way non‐rebreathing valve, with expired air passing through flexible tubing into a mixing chamber. Expired volume was measured using a ventilometer (KL Engineering Sunnyvale, CA) and mixed expired O_2_ and CO_2_ contents were analyzed by rapidly responding gas analyzers (S‐3lA/II and CD‐3A analyzers, Ametek, PA). The gas analyzers were calibrated immediately prior to each test using commercially prepared gas mixtures and the ventilometer was calibrated prior to each test using a standard 3 L syringe. The pulmonary oxygen uptake (VO_2_) was calculated each 15 sec during exercise and the highest VO_2_ at peak exercise (RPE 17) averaged over 30 sec and was termed peak VO_2_ (VO_2peak_). When exercising at RPE‐17, participants were found it difficult to maintain the requested cadence of 50–70 rpm. During the post‐training tests, participants were not given any time/workrate or physiological feedback including not being informed when they had reached their “pre‐training” workrate. This minimized any potential impact of differing motivation to exceed their pre‐training test.

### Blood sampling and analysis

A 20‐gauge catheter was inserted into an antecubital vein, attached to a 30 cm extension tube; the participant then rested supine for 10 min before a resting blood sample was obtained. Blood was sampled during cycling exercise in the final 15 sec of the last exercise workrate that corresponded to RPE 17. Two microliter of whole blood was sampled in a heparin‐coated syringe and analyzed immediately for plasma [K^+^] using an automated analyzer (Rapid point 405, Siemens Medical Solutions and Diagnostics, NY). To enable comparisons after training, the rise in [K^+^] during exercise above rest (∆[K^+^]_v_) was calculated and normalized to cumulative work (∆[K^+^]_v_.work^−1^ ratio) and expressed as nmol.L^−1^.J^−1^ (McKenna et al. 1993).

### Intense interval training

Participants trained under full supervision, three times per week for 12 weeks, with all training conducted on a mechanically braked cycle ergometer (Monark 868, Vansbro, Sweden). A standardized 3 min warm up followed by 1 min of passive rest on the cycle ergometer was conducted prior to every training session. The training was based on a previous protocol (Wisloff et al. 2007) and comprised four bouts of 4‐minute exercise intervals performed at an intensity corresponding to 90–95% HR_peak,_ interspersed by 4 min intervals; during each interval participants performed active recovery, cycling at 50–60% HR_peak_. Each training session was followed by a 5 min cool down. Throughout the training session heart rate was recorded by a heart rate monitor (RS800sd, Polar Electro Oy, Kempele, Finland). Progressive overload was implemented into the training program, by increasing the workrate as required to ensure that participants reached their target HR. Participants in the control group were asked to continue with their regular daily activities for the 12 week period. After this they also received 12 weeks of IIT, as an incentive to assist recruitment into the study and then to remain in the CON group for the entire 12 week period. No post‐training measurements were conducted on the CON group following this additional period as it would have been unreasonable to ask the participants in the CON group to undergo a greater number of invasive measurements and exercise tests than the IIT group.

### Resting muscle biopsy and single fiber separation

A resting muscle biopsy was taken prior to commencement of the training program and at 48–72 h following completion of the final training session. In the control group, consent for the post‐intervention biopsy could only be obtained from five participants. After an injection of a local anesthetic into the skin and fascia (Xylocaine 1%, AstraZeneca, Australia) a small incision was made and a *vastus lateralis* muscle sample was taken using a biopsy needle with suction. The muscle was rapidly blotted on filter paper to remove excess blood and ~15 mg of muscle was placed in a petri dish on ice coated with paraffin oil for single fiber isolation. The remaining muscle was immediately frozen in liquid nitrogen and stored at −80°C until analysis of [^3^H]ouabain‐binding site content and whole muscle homogenate NKA isoforms via western blots. Approximately 30–40 single fiber segments (~3–5 mm in length) were separated from the fresh muscle under a dissecting microscope using jeweller's forceps as previously described (Murphy 2011). All individual fiber segments were placed in separate microfuge tubes, containing 10 μL of 1× solubilizing buffer (0.125 mol/L Tris‐HCI, 10% glycerol, 4% SDS, 4 mol/L urea, 10% mercaptoethanol and 0.001% bromophenol blue, pH 6.8) diluted 2:1 with 1× Tris^.^Cl (pH 6.8) and stored at −80°C until western blotting analyses. We were unsuccessful in obtaining Type II fibers from three of the eight participants, thereby the sample size for the isoform measurements was reduced in Type II fibers.

### Whole muscle homogenate preparation

A whole muscle homogenate was prepared as described earlier (Murphy et al. 2006). Briefly, a small portion of whole muscle (15–30 mg) was accurately weighed and homogenized on ice (1:20 wt:vol) in a Na‐EGTA solution (165 mmol/L Na^+^, 50 mmol/L EGTA, 90 mmol/L HEPES, 1 mmol/L free Mg^2+^ (10.3 mmol/L total Mg^2+^), 8 mmol/L total ATP, 10 mmol/L creatine phosphate, pH 7.10) with a protease inhibitor cocktail (PIC, Complete; Roche Diagnostics, Sydney, Australia). The homogenate was then diluted to 33 μg muscle wet weight using a 3× SDS solution (0.125 mol/L Tris‐HCI, 10% glycerol, 4% SDS, 4 mol/L urea, 10% mercaptoethanol and 0.001% bromophenol blue, pH 6.8). Finally, samples were further diluted to 2.5 μg wet weight muscle.μL^−1^ using 3× SDS solution diluted 2:1 with 1× Tris^.^Cl (pH 6.8).

This homogenate served two purposes. Firstly, western blots were performed on whole muscle homogenates to determine whether any training effects that might be detected in muscle single fibers could also be detectable in a whole muscle homogenate. Second, a small portion of the homogenate from each sample was used to generate a four‐point calibration curve. This calibration curve contained ~2 mL of mixed homogenate and when each gel was run an aliquot from this calibration curve was loaded on every gel in the single fiber analyses. This provided knowledge of the linear relationships and appropriate upper and lower detection limits for the samples being measured for each NKA isoform. The calibration curve did not reach complete upper limit saturation, however, all samples fell within the limits of our calibration curve. On each gel the calibration curve showed good linearity which the vast majority of gels for each NKA isoform averaging an *r* ^2^ between ~0.98 and 0.99. The range was between *r* ^2^ 0.93 and 1.0.

### Western blotting

Western blots were performed to determine the NKA isoform protein abundance in single skeletal muscle fiber segments. In isolated fibers from five of the eight participants, individual fibers were divided and allocated across two gels allowing for the analysis of multiple isoforms in the same fiber, as previously described (Wyckelsma et al. 2016). Single fiber data were only included from participants when both pre‐ and post‐training fibers of the same fiber‐type could be collected from the biopsy samples; hence a different number of participant (*N*) and fiber numbers analyzed (*n*) are presented for the different isoforms.

The western blotting technique has been described previously (Murphy 2011; Wyckelsma et al. 2015). Briefly, single fibers were loaded onto gels with calibration curves derived from addition of graduated amounts of whole human muscle homogenate. Denatured protein samples were separated on a 26‐well, 10% or 4–15% Criterion TGX Stain Free gel (Bio‐Rad Laboratories, Hercules, CA) and electrophoresis run for 45 min at 200 V. Images of the Stain Free gels were obtained following UV activation of the gel using a Stain Free Imager (BioRad). From these gels, the abundant muscle protein, myosin was used as an indicator of total protein (Murphy and Lamb 2013). Using a wet transfer protocol, protein was transferred to nitrocellulose membrane at 100 V for 30 min. Membranes were incubated in Miser solution (Pierce, Rockford, IL) and following quick washes in milli‐Q water, blocked in 5% skim milk powder in Tris‐buffered saline‐Tween (TBST). Membranes were cut into three portions (>120 kDa, 120–70 kDa, <70 kDa) and each section was incubated with antibodies diluted in 1% bovine serum albumin in phosphate‐buffered saline with 0.025% Tween and 0.02% NaN_3_. Details of antibodies are: NKAα_1_ (a6F, Developmental Studies Hybridoma Bank (DSHB), mouse, monoclonal, 1:750), NKA α_2_ (07‐674, Millipore, rabbit, polyclonal, 1:500), NKA β_1_ (MA3‐930, Affinity Bioreagents, mouse, monoclonal, 1:500), Myosin Heavy Chain IIa (MHCIIa) (A4.74, DSHB, mouse, monoclonal, IgG, 1:200), Myosin Heavy Chain I (MHCI) (A4.840, DSHB, mouse, monoclonal, IgM, 1:200). Membranes were incubated overnight at 4°C and 2 h at room temperature, all with rocking. After washing and incubating with a secondary antibody and following TBST washes, the membrane was coated with chemiluminescent substrate (West Femto, ThermoScientific, IL). Images were taken using Image Lab software (Bio‐Rad). The positions of molecular mass markers were captured under white light prior to chemiluminescent imaging without moving the membrane. Membranes were then washed in TBST and re‐probed using a different antibody with a different animal host.

Each single fiber gel contained 10 Pre‐and 10 Post‐training fibers; when loading fibers it was unknown whether these fibers expressed the MHC I or II isoform and thus an uneven number of Type I and II fibers from before and after training were loaded, in some instances there were no Type II fibers on a gel and thus the sample size for type II fibers is reduced compared to Type I fibers. On homogenate gels, 4 μL (10 μg muscle wet weight) of homogenate was loaded in each lane. Single fiber NKA isoform abundance was measured in samples from all eight participants in the IIT group but was not measured for the CON group. Whole muscle homogenate samples from before and after IIT and before and after CON group were run on their own gel each with a 3–5 point calibration curve ranging from ~3 to 32 μg muscle wet weight. NKA isoform abundance was measured in whole muscle homogenate from eight participants from the IIT group and from four participants in the CON group.

### Western blotting analysis

For data analysis, the density of a given protein was obtained for each muscle sample and expressed relative to the calibration curve and then normalized to the total protein of their respective lane, which was also expressed relative to its standard curve. This allowed us to determine the relationship between total protein density on the stain free gel and our proteins of interest. For single fiber analysis, the linearity of each gel showed a minimum *r* ^2^ ≥ 0.9465 and for homogenate gels the linearity showed *r* ^2^ ≥ 0.9650.

We had difficulties obtaining consistent data for the β_2_ and β_3_ isoforms, as previously reported (Wyckelsma et al. 2015, 2016).

### [^3^H]ouabain‐binding site content

Approximately 20 mg of muscle was used to measure the [^3^H]ouabain‐binding site content, as previously described as a measure of whole muscle NKA content in human muscle (Nørgaard et al. 1984; Petersen et al. 2012). Briefly, each sample was washed for 2 × 10 min at 37°C in vanadate buffer (250 mmol/L sucrose, 10 mmol/L Tris‐HCl, 3 mmol/L MgSO_4_, 1 mmol/L NaVO_4_; pH 7.3). Following washing, samples were incubated for 2 h at 37°C in vanadate buffer with the addition of [^3^H]ouabain (1.6 μCi.mL^−1^ and 10–6mol/L, PerkinElmer Inc., Boston, MA). The muscle was then placed in ice‐cold vanadate solution for 4 × 30 min to remove any unbound [^3^H]ouabain. The muscle samples were divided into four ~5 mg pieces, which were individually blotted on filter paper and weighed before being soaked in 500 μL 5% trichloroacetic acid and 0.1 mmol/L ouabain for ~20 h. Following this, 2.5 mL of scintillation cocktail (Opti‐Fluor, Packard, PerkinElmer Inc., Boston, MA) was added before liquid scintillation counting of [^3^H]ouabain. The content of [^3^H]ouabain‐binding site content was calculated on the basis of the sample wet weight and specific activity of the incubation buffer and samples, and expressed as pmol.g wet wt^−1^. The final [^3^H]ouabain content was then calculated accounting for unspecific binding, correction factor for impurity of [^3^H]ouabain, loss of bound [^3^H]ouabain during washout and incomplete saturation, as previously described (Nørgaard et al. 1984). The muscle [^3^H]ouabain‐binding analysis was conducted on eight participants from the IIT group and five from the CON group.

### Statistical analysis

The single fiber NKA isoform data was not normally distributed and therefore was log‐transformed before analysis, while all other data was normally distributed and were analyzed as raw data. A univariate nested model analysis (linear mixed model) was performed to detect any fiber‐type specific training differences in single fibers. The effects of IIT on physical characteristics (body mass, body mass index, blood pressure), were measured using a two‐tailed paired t‐test. Due to hypothesized unidirectional change after training based on findings from previous studies in young adults (McKenna et al. 1993; Nielsen et al. 2004; Mohr et al. 2007), the effects of IIT on peak exercise variables (workrate, duration to RPE‐17, VO_2__peak_, heart rate, plasma [K^+^]), as well as on muscle [^3^H]ouabain‐binding were analyzed using a one‐tailed paired t‐test with IIT and CON groups analyzed individually. The CON exercise data pre and post was analyzed using a two‐tailed paired t‐test along with all whole muscle homogenate data from both groups. Calculated effect size using Cohen's *d* are presented, Cohen's conventions for effect size were adopted for interpretations, where 0, 0.2, 0.5, and 0.8 are considered trivial, small, moderate and large, respectively (Cohen 1988). Moderate to large effect size signify a functional effect of an intervention. All statistics were performed on SPSS statistics version 22.0. All data are presented as mean ± SD. Significance was set as *P* < 0.05.

## Results

### Compliance to training and adverse responses occurring from IIT

Two of the 15 commencing participants discontinued the training program, the first due to ill health unrelated to this study and the other due to an abnormally high blood pressure response to exercise; their data has not been included here. All other participants completed a minimum 83% (30 out of 36) of training sessions. Testing and training were generally well tolerated by these older adults. However, in the first 2 weeks of training five out of ten participants experienced mild vasovagal episodes during the course of training; with no further subsequent incidents.

### Physical characteristics

IIT had no effect on resting systolic blood pressure (Pre, 134.0 ± 17.0 vs. Post, 125.6 ± 10.3 mmHg, *P* = 0.13, *d* = −0.7) but had no effect on resting diastolic blood pressure (Pre, 80.5 ± 8.1 vs. Post, 80.0 ± 1.6 mmHg) or body mass (Pre, 74.7 ± 9.3 vs. Post, 74.3 ± 10.2 kg). There were no changes in CON for any of body mass (Pre, 75.2 ± 15.8 vs. Post, 74.9 ± 15.7 kg), systolic (Pre, 132.0 ± 12.0 vs. Post, 136.0 ± 16.0 mmHg) or diastolic blood pressure (80.3 ± 10.2 vs. 82.6 ± 7.5 mmHg).

### Exercise performance and plasma [K^+^]

IIT increased each of WR_peak_ (25 ± 23%), total work (J) (60 ± 46%), VO_2peak_ (16 ± 12%), and HR_Peak_ (6 ± 8%), (*P* < 0.05) and tended to increase exercise time until a RPE‐17 was attained (*P* = 0.057) during the incremental test (Table 1). There were no changes in CON for any performance measures (Table 1).

**Table 1:** The effects of 12 weeks of intense interval training (IIT) in elderly humans aged between 65 and 76 years, on performance and physiological variables including peak potassium, during incremental exercise continued until a RPE‐17

Variable	Group	Pre	Post	*P* value	Cohen's d
WR_peak_ (W)	IIT	145.0 ± 49.5	181.2 ± 52.4	*P* = 0.01	1.6
	CON	142.0 ± 46.4	147.1 ± 40.2	NS	0.0
Work (J)	IIT	43,725 ± 21,282	70,050 ± 31,834	*P* = 0.001	2.1
	CON	47,914 ± 24,408	47,486 ± 24,834	NS	−0.0
Time to RPE‐17 (min)	IIT	7.3 ± 3.7	9.4 ± 4.5	NS (*P* = 0.057)	0.6
	CON	9.6 ± 3.1	9.7 ± 3.1	NS	0.2
HR _peak_ (*b*.min^−1^)	IIT	136.2 ± 16.4	144.3 ± 14.4	*P* = 0.03	0.7
	CON	141 ± 11	142 ± 14	NS	0.5
VO_2peak_ (mL.kg^−1^.min^−1^)	IIT	24.7 ± 5.4	28.7 ± 5.1	*P* = 0.002	1.4
	CON	23.6 ± 5.3	23.8 ± 5.3	NS	0.0
[K^+^]_v peak_	IIT	4.74 ± 0.41	5.23 ± 0.57	NS (*P* = 0.07)	0.0
(mmol.L^−1^)	CON	4.88 ± 0.33	4.90 ± 0.42	NS	0.0
∆[K^+^]_v_.work^−1^	IIT	21.4 ± 10.6	17.4 ± 4.5	NS	0.4
(nmol.L^−1^.J^−1^)	CON	22.3 ± 10.9	21.1 ± 14.7	NS	−0.0

Data is expressed as mean + SD; *n* = 8 IIT, *n* = 7 CON.

Following IIT, the peak exercise plasma [K^+^]_v_ at RPE‐17 tended to be higher than pre‐training (10%, 0.49 mmol/L, *P* = 0.056, Table 1), with no differences observed in CON (Table 1). The ∆[K^+^]_v_.work^−1^ ratio did not change following either IIT or CON (Table 1).

### Muscle [^3^H]ouabain‐binding site content

**Figure 1:**
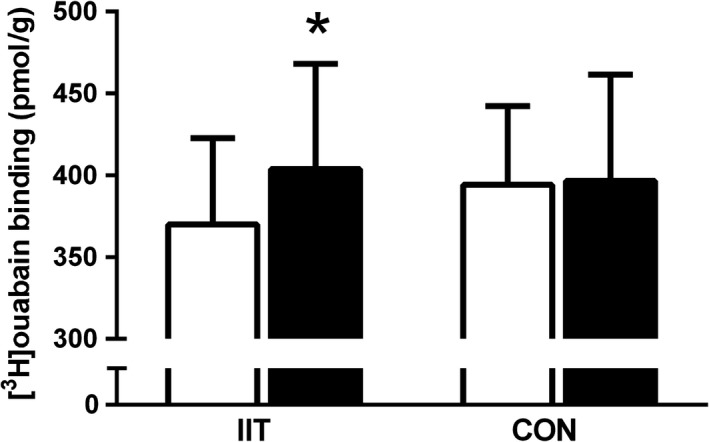
The effects of 12 weeks of intense interval training (IIT) in elderly humans aged between 65 and 76 years, on the skeletal muscle Na^+^,K^+^‐ATPase (NKA) content, measured by [^3^H]ouabain‐binding site content before (Pre, open bars) and after IIT‐training (Post, filled bars) IIT or control (CON). Data presented as mean ± SD; *n* = 8 HIT,* n* = 5 CON. *IIT Post‐Training greater than Pre Training, *P* < 0.05.

Gender had no effect on [^3^H]ouabain‐binding site content in older adults (Male 373.8 ± 57.4 vs. Female 344.1 ± 57.1 pmol.g^−1^, *P* = 0.3). Following IIT the muscle NKA content increased by 11% (*P* = 0.03, *d* = 0.8), with no changes observed in CON (Fig. 1).

### NKA isoform abundances

**Figure 2:**
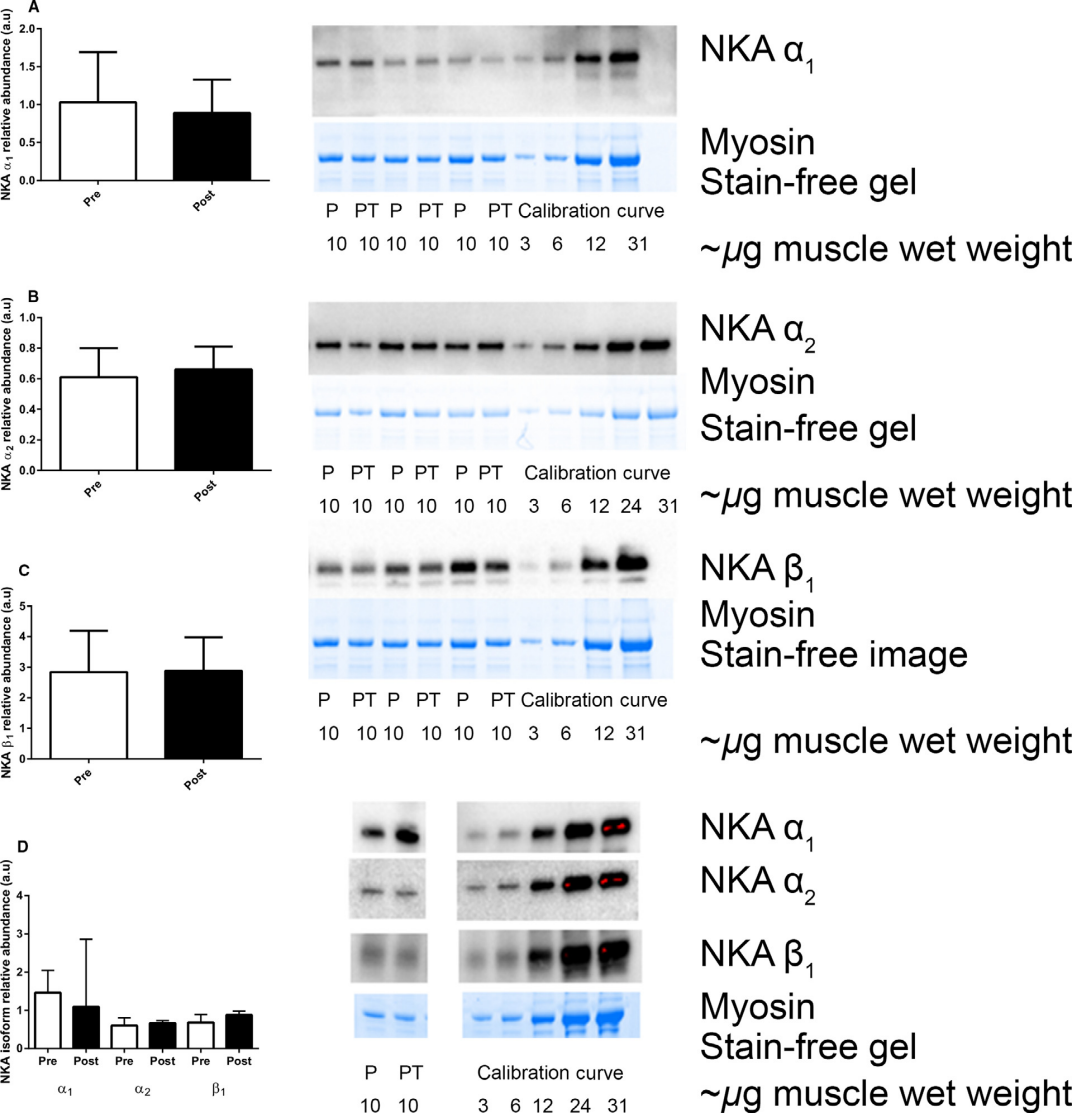
The effects of 12 weeks of intense interval training (IIT) in elderly humans aged between 65 and 76 years, on the Na^+^,K^+^‐ATPase (NKA) isoform abundances in muscle homogenates. For each panel, the graph on the left shows the mean + SD data and the representative western blot including calibration curves 3–31 μg of wet weight tissue and the loading control of the abundant muscle protein myosin in the Stain Free gel image with homogenate samples from Pre‐ (P) and Post‐Training (PT) is shown on the right. (A) NKA α1, (B) α2 and (C) β1 isoform abundances, Pre (open bars) and Post‐Training (closed bars) (D) NKA α1, α2 and NKA β1 isoform abundances in CON. The α1 and α2 isoforms migrated at ~100 kDa and the β1 isoform between ~50 and 55 kDa. Data is normalized to each lane of total protein in the Stain‐free image and then to the calibration curve. Data is mean + SD,* n* = 8 IIT.

Analyses in whole muscle homogenates revealed no changes in the NKA α_1_, α_2_ or β_1_ isoforms following 12 weeks of IIT or CON (Fig. 2). Effect size calculations revealed a moderate effect of IIT on the α_2_ isoform (*d* = 0.6).

**Figure 3:**
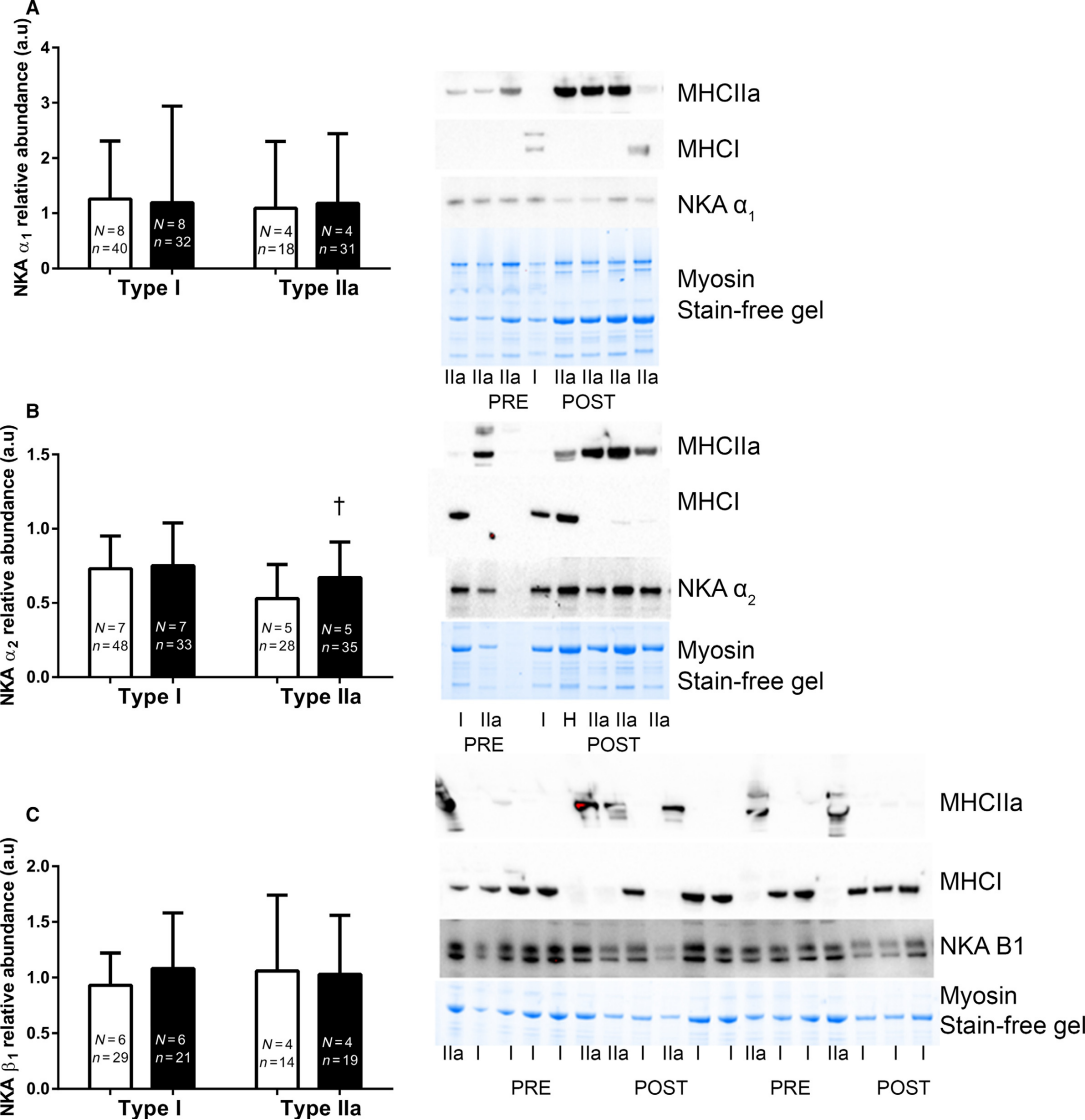
The effects of 12 weeks of intense interval training (IIT) in elderly humans aged between 65 and 76 years, on the Na^+^,K^+^‐ATPase (NKA) isoform abundances in Type I and Type II muscle fibers. For each panel the mean + SD data is shown on the left and the representative blots with Type I and Type IIa fibers from before (Pre) and after (Post) HIT shown on the right for each of the α_1_, α_2_ and β_1_ isoforms, respectively, with samples from. (A) NKA α_1_, (B) α_2_ and (C) β_1_ isoform abundances, Pre Type I (open bars), Post Type I (closed bars), Post Type II (open bars), Post Type II (closed bars). For the representative images, the fiber‐type is identified using antibodies specific to MHCI or MHCIIa, the NKA isoform is shown and the abundant muscle protein, myosin is shown on the Stain Free gel image, which indicates the relative amount of tissue loaded in each lane. Analysis involved determining the density of the specific NKA bands and expressing relative to the total protein obtained from the entire StainFree gel. The α_1_ and α_2_ isoforms migrated at ~100 kDa and the β_1_ isoform between ~50 and 55 kDa. Each lane is marked as either a Type I (I) or Type II (IIa) muscle fiber. The number of subjects from whom fibers were collected (*N*) and the total number of fibers analyzed (*n*) are shown on each graph. All data was analyzed by a univariate nested model analysis (linear mixed model).

When NKA isoform abundances were measured in single fiber segments, no changes were seen in the NKA α_1_ or β_1_ abundance following IIT in either Type I or II fibers (α_1_ *P* > 0.05, *d* = 0.2 and 0.4, Type I and II respectively, β_1_ *P* < 0.05, *d* = 0.4 and 0.0). The α_2_ abundance was unchanged after IIT in Type I fibers (*P* > 0.05, *d* = 0.1), but was increased by 30% in Type II fibers (*P* < 0.05, *d* = 0.5, Fig. 3).

## Discussion

We report that 12 weeks of IIT increased muscle NKA content by 11% in older adults and that fiber‐type specific upregulation occurred for the NKA α_2_ isoform, being elevated by 30% in Type II fibers after IIT. No changes in NKA isoforms occurred in Type I fibers after IIT. In addition no increases were detected in the NKA the α_1_, α_2_ or β_1_ isoform abundances in whole muscle homogenates after training. While we also found that IIT increased VO_2peak_ by 16% and peak workrate by 11%, there was no significant reduction in the rise in venous [K^+^] relative to work performed during incremental exercise, suggesting that at least in antecubital venous blood, IIT did not improve circulating K^+^ homeostasis. Adverse responses to IIT in some individuals during the initial 2 weeks indicate that IIT should however, be implemented with appropriate caution in healthy older adults.

### Intense interval training increases muscle NKA content and isoforms

We show for the first time that 12 weeks of IIT increased muscle [^3^H]ouabain‐binding in healthy older adults. This demonstrates that the NKA remains highly adaptable in skeletal muscle in an aged cohort, and that this occurs even in a “low volume” (i.e., 4 × 4 min, totaling 16 min per session) training protocol as utilized here. This increase in NKA content is consistent with an earlier finding in a cross‐sectional study that reported older adults who had been active for 10–12 years had a 30–40% greater NKA content than inactive older adults (Klitgaard and Clausen 1989). Our data supports the cross‐sectional data showing for the first time in elderly individuals undertaking a longitudinal training program, that there was an 11% gain in NKA content after IIT, which typically reported in studies with healthy young adults after various forms of physical training (McKenna et al. 1996). We also note, that gender of the participants had no effect on NKA content and thus their training responses, this is the first time that this has been reported in older adults in previous findings in healthy young adults (Murphy et al. 2007).

A novel finding was the fiber‐specific upregulation of the NKA α_2_ isoform after IIT in older adults, being increased in Type II fibers only; this is the first time an upregulation of α_2_ in a fiber‐specific manner has been seen after training in any age group. In rodent muscle, the α_2_ may represent ~80–85% of all α isoforms (Hansen 2001; Clausen 2003) and is particularly important in regulation of Na^+^/K^+^ exchange and membrane potential during muscle contractions (He et al. 2001; Radzyukevich et al. 2013). Furthermore, the α_2_ is typically upregulated in muscle in response to high‐intensity exercise training in healthy young adults, when measured in muscle lysates (Nielsen et al. 2004; Mohr et al. 2007; Bangsbo et al. 2009; Thomassen et al. 2010), although not always (Aughey et al. 2007; Gunnarsson et al. 2013; Wyckelsma et al. 2015). In aged muscle, Type II fibers undergo the greatest atrophy (Evans and Lexell 1995) and exhibit reductions in specific force compared to young adults (Lamboley et al. 2015). The increased α_2_ abundance specifically in Type II fibers after IIT may have important implications for maintenance of muscle function in the aged. This is based on research in skeletal muscle α_2_ knockout mice (skα_2_^−/−^) (Radzyukevich et al. 2012), which exhibited a markedly impaired treadmill incremental running performance, lower tetanic force and more rapid fatigue, compared with wild‐type mice (Radzyukevich et al. 2012). In rat isolated skeletal muscle, blocking 26% of the NKA with ouabain induced a substantial impairment of contractile force when exposed to high K^+^ solutions (Clausen and Everts 1991). We recently found no difference in α_2_ abundance in single muscle fibers and homogenate between old and young humans (Wyckelsma et al. 2016). Thus, the increase in α_2_ with IIT does not appear to be a compensatory response to an age‐related deficit of NKA isoforms, as suggested in rat muscles (Ng et al. 2003). Rather, the increased α_2_ in Type II fibers and [^3^H]ouabain‐binding site content with IIT suggests upregulation of NKA α_2_ may assist the muscle Type II fibers to undertake repeated contractions and attenuate muscle fatigue in these older adults. This is consistent with improved contractility of aged muscle and enabling the completion of simple everyday tasks requiring repeated muscle contractions in older adults. Whether the excitability of Type II fibers during muscle contractions is improved in the aged following training is of importance and remains to be determined.

An interesting finding was the lack of change in either the α_1_ or β_1_ isoforms in the single fibers or homogenates following IIT in older adults. Possible rationales for the lesser adaptability of the α_1_ and the β_1_ isoforms with training, are that the purported importance of the α_1_ is primarily under rest conditions (He et al. 2001) and an existing overabundance of the β isoforms relative to the α isoforms in muscle (Lavoie et al. 1997). However, this finding regarding α_1_ and β_1_ is also not unexpected, given the inconsistencies in the reported responses of these NKA isoforms to intense exercise training in young adults. For example, of nine studies in humans that investigated the effects of exercise training on skeletal muscle homogenate/lysate NKA α_1,_ α_2_ and β_1_ isoforms in young adults, an increase in α_1_ was reported in only three (Nielsen et al. 2004; Green et al. 2008; Iaia et al. 2008), with α_1_ unchanged in the remaining six studies (Aughey et al. 2007; Mohr et al. 2007; Bangsbo et al. 2009; Thomassen et al. 2010; Gunnarsson et al. 2013; Wyckelsma et al. 2015). For the α_2_ isoform, increases were reported after training in only five of these studies (Nielsen et al. 2004; Mohr et al. 2007; Green et al. 2008; Bangsbo et al. 2009; Thomassen et al. 2010) being unchanged in the other four, whilst increases in the β_1_ isoform were only reported in three of these nine studies (Mohr et al. 2007; Green et al. 2008; Wyckelsma et al. 2015) being unchanged in the other six. Recently, resistance training following a period of muscle disuse resulted in an upregulation of α_1_ in Type II fibers and α_2_ in Type I fibers, but with no changes detected for either isoform in a crude homogenate (Perry et al. 2016). There does not appear to be a clear link between the type of training or in the training status of individuals in the adaptability of these NKA isoforms in younger adults or whether these analyses utilized whole muscle homogenates or spun muscle lysates.

Our findings revealed differences in the relative changes in the [^3^H]ouabain‐binding site content and the α_2_ abundance after IIT. After IIT, the NKA content measured by [^3^H]ouabain‐binding assay was increased by 11%, the α_2_ abundance detected in Type II fibers increased by 30%, whilst the α_2_ abundance in whole muscle homogenates and in Type I fibers were unchanged. This firstly suggests that the [^3^H]ouabain‐binding assay may be more sensitive in detecting changes in mixed muscle analyses that are restricted to only one fiber‐type, than western blotting conducted with whole muscle homogenates; and secondly, that the [^3^H]ouabain‐binding assay can detect changes restricted to one fiber‐type if this change is relatively large. Numerous studies investigating the effects of training, aging, injury and disease on NKA content have reported that the relative increases in the protein abundance of α_2_ measured in muscle homogenates were greater than those when measured with [^3^H]ouabain‐binding site content, with similar proportional increases reported only five times out of ten studies in the literature (Clausen 2013). Our findings show that our [^3^H]ouabain‐binding responses were greater than the α_2_, the opposite to what is normally shown, however this may be simply related to differences in western blot techniques, including the use of a whole homogenate versus lysates. Regardless, this study has shown that the NKA content remains highly adaptable to physical training with age and further expands on our previous work where we speculated that physical activity levels, rather than age, directly affected muscle NKA content (Wyckelsma et al. 2016). This is also consistent with our earlier observation in patients with osteoarthritis that physical activity may play an important role in preserving NKA content and α_2_ abundance in the aged (Perry et al. 2013).

### Physiological adaptations and other practical implications

A further novel finding was the effectiveness of IIT for improving important functional outcomes in healthy older adults. The IIT protocol was effective at increasing each of the VO_2peak_, WR_peak_ and HR_peak_ of older adults and also tended to increase each of exercise time to a RPE17, systolic blood pressure and [K^+^]_v peak_. We found no differences in ∆[K^+^]_v_.work^−1^ ratio, suggesting that the tendency for higher peak [K^+^]_v_ at the end of exercise after IIT was simply a function of being able to attain a higher WR_peak_ after training. This finding differs from other training studies in young adults, that have demonstrated reduced ∆[K^+^].work^−1^ ratio following training, when [K^+^] was measured during sprint or high‐intensity exercise (McKenna et al. 1993, 1997; Harmer et al. 2006) or during submaximal exercise (Nielsen et al. 2004), although this was not always the case (McKenna et al. 1997). Thus, despite the improved exercise performance and upregulation of the [^3^H]ouabain‐binding and NKA α_2_ in Type II fibers after IIT, no change was seen in the [K^+^]_v_.work^−1^ ratio. One possible interpretation is that there was no improvement in the in‐vivo K^+^ regulation in these older individuals after training; however, several factors will have affected these [K^+^] findings and may make this interpretation invalid. First, blood was sampled from the antecubital vein, draining the relatively inactive forearm muscles during exercise, with the [K^+^]_v_ being substantially lower than in arterial blood or in venous blood draining the active musculature (Lindinger 1995; Sejersted and Sjøgaard 2000). Measurement of the femoral venous or arterial [K^+^] rather than antecubital venous [K^+^] would have been preferred. However, we judged that in these elderly participants, these more demanding procedures were impractical, may have been difficult to justify ethically, and may have compromised recruitment and retention. Second, for safety reasons the test was only continued until a RPE 17 (“very hard”) was achieved, and thus the exercise was not truly maximal, which would underestimate the peak exercise workrate and plasma [K^+^] (Medbø and Sejersted 1990). Pre‐training, participants reached a HR_peak_ of ~136 b.min^−1^ during exercise, estimated at 88–92% of the age‐predicted maximal heart rate (220‐age), whereas after training, a HR_peak_ of ~144 *b*.min^−1^ was attained, estimated to correspond to between 93% and 100% of the age‐predicted maximal heart rate. This suggests that after IIT, older adults were able to work at a higher workrate and closer to their age‐predicted maximal HR. It is unlikely that this results simply from a greater motivation to exercise harder after training. From a practical aspect, the implementation of IIT for the general population of older adults should be performed with great caution due to potential adverse responses during the first few weeks of training. In this study, 50% of the participants exhibited mild vasovagal episodes during the first 2 weeks of training. It is recommended that those who wish to perform IIT should have had recent exposure to at least regular moderate intensity exercise, and have appropriate supervision and monitoring during and after each exercise session (Levinger et al. 2015).

## Conclusions

IIT upregulated NKA α_2_ isoform in Type II fibers and this coincided with increases in whole muscle NKA content measured by [^3^H]ouabain‐binding site content. These findings indicate that skeletal muscle from elderly individual remains highly adaptable to exercise training with respect to muscle NKA. Further research is required to determine the functional significance of this increase in NKA α_2_ with training. Whilst IIT improved exercise performance in older adults, it should be implemented with caution due to potential adverse responses.

## Conflict of Interest

The authors declare no conflict of interest.
